# The effect of dietary energy and protein levels on body weight, size, and microflora of ostrich chicks

**DOI:** 10.1007/s11250-017-1480-8

**Published:** 2017-11-24

**Authors:** Tahereh Nikravesh-Masouleh, Alireza Seidavi, Magdalena Kawka, Mohammad Dadashbeiki

**Affiliations:** 1 Department of Animal Science, Rasht Branch, Islamic Azad University, Rasht, Gilan 41335-3516 Iran; 2 Department of Genomics, Institute of Genetics and Animal Breeding PAS, ul. Postępu 36A, 05-552 Jastrzębiec, Poland; 3 Department of Veterinary Medicine, Rasht Branch, Islamic Azad University, Rasht, Gilan 41335-3516 Iran

**Keywords:** Energy, Protein, Body size, Microbial population, Ostrich chickens

## Abstract

The aim of this study was to evaluate the effect of different dietary energy and protein supplements on performance, weekly body sizes or body frame size, and microbiota of ostrich chicks during 2–9 weeks of age. Two metabolic energy levels of 2400 and 2600 kcal/kg live weight and three protein levels of 20, 22, and 24% were used. A total of 36 ostrich chickens (*Struthio camelus*) of the blue and black African breed were used. Body weight, 12 body measurements (i.e., circumference of the head, neck, breast, abdomen, thigh, body height, length of tail, list the other 5 here) and excretion of microbial population (*Escherichia coli*, *Coliforms bacteria*, and *Lactobacillus bacteria*) were measured. Mean body weight in each week of the experiment was generally the lowest when chicks were offered 2600 kcal/kg dietary energy and 24% protein. Of the 12 body measurements, the breast, abdomen, and thigh circumference and also body length were greater at the lower energy (2400 kcal/kg) and higher protein (24%) levels. Total *Aerobic* bacteria excretion was generally lower in response to the diet containing the higher level of energy. We conclude that ostrich chickens during 2–9 weeks of age can grow on diets that contain lower energy levels.

## Introduction

Ostriches are well adapted to the environmental conditions, and interest in rearing them commercially is growing in many countries of the world (Cloete et al. 2012). The great interest in ostrich breeding has led to an increase in the demand for information about this bird, especially its maintenance and nutritional requirements (Brand et al. 2015) and the potential for genetic improvement (Kawka et al. 2010, 2012a, 2012b). Nutrition is one of the parameters that ostrich producers have the most influence. The two main nutrients in most livestock diets are energy and protein. Energy has the greatest influence on feed intake. Protein with energy is a macronutrient, and both comprise the bulk of the digestible matter contained in animal diets. Knowledge of nutritional requirements during the various stages of growth, development, and production of the ostrich are vital (Bovera et al. 2014). The nutrient requirements of an ostrich depend on its stage of growth (Brand and Olivier 2011). Cooper et al. (2005) stated that most ostrich performance problems relating to fertility can be traced back to an inadequate breeder diet. Despite some recent reports on ostrich nutrition (e.g., Poławska et al. 2014; Jóźwik et al. 2015), the knowledge in this area is still meager as compared to other monogastrics including broilers and pigs. A global literature search yield publications related to feeding ostriches using feed of different origin and concentration of energy (Viljoen 2011; Brand et al. 2014; Karimi-Kivi et al. 2015; Tasirnafas et al. 2014, 2015). However, research evaluating the performance of commercial ostrich chicks fed with diets differing in protein and dietary energy is still lacking. Therefore, in this study, the effect of two different levels of metabolizable energy (2400 and 2600 kcal/kg) and three different levels of protein (20, 22, and 24%) in the diets of ostrich chicks was evaluated in terms of their performance and changes in weekly body size and microbiota parameters.

## Material and methods

### Animals, housing, and management

This research was conducted from the 2nd–9th weeks (56 days) of age at an ostrich farm located in Guilan, Iran. Thirty-six blue and black ostrich chicks (*Struthio camelus*) with equal weight were selected. The selected ostrich chicks were housed in land cages 5 × 3 m (two ostrich chicks/land cages). Temperature, humidity, lighting, health programs, and other management programs were applied based on standard protocols. The chicks were vaccinated following the standard vaccination schedule. Birds received a natural regime for lighting, temperature, and humidity throughout the study period. Ethics approval for the animal trials was obtained from the Animal Ethics Committee, Rasht Branch, Islamic Azad University, Rasht, Iran.

### Treatments and experimental diets

The 36 ostrich chicks were allocated to six treatments with three replicates per treatment and also 2 ostrich chicks per replicate. Treatments T1 to T3 were given a diet with 2400 kcal/kg of metabolizable energy and with 20, 22, and 24% of dietary protein. Treatments T4 to T6 were comprised of a diet of 2600 kcal/kg metabolizable energy with 20, 22, and 24% of dietary protein. The ingredients and composition of the diets used is shown in Table 1. Food and drinking water for all ostrich chickens were offered ad libitum.

**Table 1:** Feed ingredients of used diets during experiment

	Treatment 1	Treatment 2	Treatment 3	Treatment 4	Treatment 5	Treatment 6
Metabolizable energy (kcal/kg)	2400	2600	2400	2600	2400	2600
Protein (%)	20.00	20.00	22.00	22.00	24.00	24.00
Ingredient						24.0
Corn	44.60	50.00	41.21	47.81	38.00	44.20
Soybean meal	29.30	29.15	35.00	30.00	39.55	36.64
Gluten meal	–	–	–	3.80	0.50	2.40
Wheat bran	17	12.14	13.00	4.00	5.00	10.00
Alfalfa meal	2.65	3.00	5.00	9.00	12.00	5.00
Oyster	1.43	2.00	1.61	1.88	1.98	–
Ca%22P%18	4.00	2.70	3.17	2.50	1.96	0.75
NaCl	0.41	0.40	0.40	0.40	0.40	0.40
Mineral mixture^1^	0.25	0.25	0.25	0.25	0.25	0.25
Vitamin mixture^2^	0.25	0.25	0.25	0.25	0.25	0.25
Anti-fungus	0.01	0.01	0.01	0.01	0.01	0.01
DL-methionine	0.10	0.10	0.10	0.10	0.10	0.10
Total	100	100	100	100	100	100
Price (rial/kg)	9088.78	8890.55	9196.41	9507.04	9317.67	9255.80

^1^Ca(IO3)2, 200.4 mg/kg; Se, 40 mg/kg; CuSO4, 3000 mg/kg; Fe2(SO4)3, 4000 mg/kg; ZnO, 14,025,667 mg/kg; MnO, 20,000 mg/kg; NaCl, 54,015 mg/kg; MgO, 2021 mg/kg; CoSO4, 80 mg/kg; S, 40,095 mg/kg

^2^Vitamin A, 1,800,000 IU/kg; vitamin B1, 300.86 mg/kg; vitamin B2, 1400 mg/kg; vitamin B3, 2000.18 mg/kg; vitamin B5, 10,000.745 mg/kg; vitamin B6, 800.66 mg/kg; vitamin B9, 300.2 mg/kg; vitamin B12, 2 mg/kg; vitamin K3, 400 mg/kg; vitamin D3, 400,000 IU/kg; vitamin E, 4000 IU/kg; vitamin H2, 4 mg/kg; vitamin C, 4000.59 mg/kg; choline chloride, 40,200 mg/kg; antioxidant, 1000 mg/kg

### Measured traits and body characteristics

During the experimental period, body weight was measured on a weekly basis using a digital balance MDS 15000AP (Mahak Co., Iran).

Body characteristics (beak round, beak circumference, head circumference, neck outset circumference, neck windup circumference, neck height, breast circumference, abdomen circumference, pelvis circumference, tail length, thigh circumference, thigh length, shank circumference, shank length, leg length, arm circumference, arm length, forearm circumference, forearm length metacarpal bone length, body length, and body height) were recorded weekly.

### Microbiota analysis

Feces of one bird from each replicate were collected under sterile conditions using a swab for further culture. Agar plates were streaked with feces and sent to the laboratory of Nutrition and Dairy Industry at Rasht Branch, Islamic Azad University. Bacterial growth and colony counts were assessed using the agar plates streaked on the site. The culture media were prepared and were poured into the petri dish 24 h before collecting samples. MRS agar (Man Rogosa Sharpe agar, 1.10660.500) to culture Lactobacilli, Eosin Metilan Blou (EMB, 1.01347.0500) to culture *Escherichia coli*, and MacConkey agar (105465.0500) to culture Coliforms was used. Nutrient agar (1.05450.0500) was used to culture total aerobic bacteria counts, respectively. The same protocols were used to characterize bacteria isolated from gastrointestinal contents and suspended in medium. A colony counter was used to count the bacteria in the petri dishes. Bacterial counts were reported as logarithmic counts of bacteria per gram of sample.

### Statistical analysis

Data were analyzed using a two-way ANOVA procedure and SAS (2003) statistical software, and GLM procedure was used. The means were compared by using DUNCAN’s multiple range. The results were considered significantly different when *P* < 0.05.

## Results

Table 2 shows the mean body weight of ostrich chicks fed with diets containing the different levels of energy and protein from the 2nd–9th weeks of age. The mean body weight in each week of the experiment was the lowest in the case of the 6th treatment (2600 kcal/kg dietary energy and 24% protein). The highest level of body weight was observed in birds offered the 2400-kcal/kg dietary energy level with 20 or 22% protein. The results for body size characteristics of birds in the 2nd and 9th weeks of age are presented in Table 3. We showed only these 2 weeks of age because the same trends of body size were observed in the intervening weeks between weeks 2 and 9. In the 2nd week, the highest level of breast, abdomen, and arm circumference, thigh and leg length, and body length and height were observed in birds offered 2400-kcal/kg dietary energy level. The remaining characteristics (beak and thigh circumference and arm length) were greater in birds offered the 2600-kcal/kg energy level. Protein levels did not significantly affect the body characteristics except leg length, where the difference between the lowest and the highest protein level was about 50%. In this period, the body length and height ranged from 54.61 to 57.61 cm and from 24.58 to 26.47 cm, respectively. In the 3rd week of age, the relationships between body size characteristics were the same. However by week 4, all studied traits were higher in birds offered the 2400-kcal/kg energy level except for beak circumference. At the 5th week of age, only neck height, thigh circumference, and arm and body length were smaller with the lower energy level. In the 6th and 7th weeks of age, most measurements were higher in birds offered the 2400-kcal/kg energy level. In the final experimental period (8th and 9th week of age), trends in the measured body characteristics were the same as for the 6th and 7th weeks. The lowest abdomen and thigh circumferences at the 9th week of age were found in birds offered the 2600-kcal/kg dietary energy level together with 20% protein. The highest abdomen circumference was observed in birds offered the 2400-kcal/kg dietary energy and 22% protein levels, while the highest thigh circumference was found in birds given the 2400-kcal/kg dietary energy and 20% protein levels. The breast circumference ranged from 45.66 to 53.08 cm. The highest body lengths and body heights were found in birds fed with 2400 kcal/kg dietary energy and 24% protein. Leg length and arm circumference were both higher in birds fed with the lowest energy level. Both body weight and body measurements were higher in birds offered with the higher protein level (22 and 24%). Analysis of excreta microbial population of ostrich chicks assessed in week 9 is given in Table 4. The presence of *Escherichia coli*, *Coliforms bacteria*, *Lactobacillus bacteria*, and total aerobic bacteria were measured in these samples. The level of *Escherichia coli* ranged from 8.20 CFU/g (4th treatment) to 8.89 CFU/g (3rd treatment). The lowest level of C*oliforms* bacteria was associated with the 4th treatment—8.36 CFU/g. Whereas, the highest level of *Lactobacillus* bacteria was observed with birds fed with 2400 kcal/kg dietary energy and 20% protein. The total aerobic bacteria count was generally lower in birds offered with the diet containing higher energy level (2600 kcal/kg).

**Table 2:** Mean body weight (±SE) of ostrich chicks at consecutive weeks of age fed with diets containing the different levels of energy and protein from the 2nd–9th weeks of age (kg/ostrich)

		Trait							
Treatment		2nd week of age (14th day of age)	3rd week of age (21st day of age)	4th week of age (28th day of age)	5th week of age (35th day of age)	6th week of age (42nd day of age)	7th week of age (49th day of age)	8th week of age (56th day of age)	9th week of age (63rd day of age)
Energy (kcal/kg DM)	2400	1.21^a^ ± 0.14	1.89^a^ ± 0.19	3.07^a^ ± 0.37	4.28^a^ ± 0.41	5.54^a^ ± 0.57	7.57^a^ ± 0.59	9.00^a^ ± 0.82	10.97^a^ ± 0.93
	2600	1.13^b^ ± 0.14	1.61^b^ ± 0.19	2.45^b^ ± 0.31	3.43^b^ ± 0.51	4.53^b^ ± 0.51	5.82^b^ ± 0.76	0.86 ± 7.24^b^	9.06^b^ ± 0.98
Protein (% in diet)	20	1.23^a^ ± 0.15	1.83^a^ ± 0.17	2.80^ab^ ± 0.40	3.91^a^ ± 0.72	5.00^a^ ± 0.45	6.68^a^ ± 1.28	7.84^a^ ± 1.19	9.72^a^ ± 1.79
	22	1.16^a^ ± 0.17	1.80^a^ ± 0.33	2.94^a^ ± 0.64	3.91^a^ ± 0.74	5.27^a^ ± 0.85	6.80^a^ ± 0.98	8.51^a^ ± 1.01	10.56^a^ ± 1.24
	24	1.12^a^ ± 0.12	1.63^a^ ± 0.15	2.55^b^ ± 0.22	3.75^a^ ± 0.50	4.84^a^ ± 0.91	6.62^a^ ± 1.27	8.00^a^ ± 1.54	9.76^a^ ± 1.64
Energy (2400)–protein (20)		1.29^a^ ± 0.10	1.92^a^ ± 0.04	3.09^ab^ ± 0.34	4.46^a^ ± 0.11	5.31^ab^ ± 0.21	7.75^a^ ± 0.22	8.70^ab^ ± 0.93	10.73^a^ ± 0.37
Energy (2400)–protein (22)		1.22^a^ ± 0.12	2.04^a^ ± 0.23	3.44^a^ ± 0.09	4.34^ab^ ± 0.53	5.79^a^ ± 0.65	7.43^ab^ ± 0.73	9.09^a^ ± 0.60	11.25^a^ ± 0.94
Energy (2400)–protein (24)		1.12^a^ ± 0.15	1.73^ab^ ± 0.12	2.70^bc^ ± 0.14	4.06^ab^ ± 0.53	5.52^a^ ± 0.81	7.55^ab^ ± 0.86	9.21^a^ ± 1.11	10.93^a^ ± 1.50
Energy (2600)–protein (20)		1.18^a^ ± 0.20	1.75^ab^ ± 0.23	2.52^c^ ± 0.22	3.37^b^ ± 0.64	4.69^ab^ ± 0.43	5.61^c^ ± 0.80	6.99^bc^ ± 0.68	8.71^b^ ± 0.92
Energy (2600)–protein (22)		1.11^a^ ± 0.15	1.57^bc^ ± 0.22	2.45^c^ ± 0.55	3.48^ab^ ± 0.74	4.75^ab^ ± 0.87	6.17^bc^ ± 0.96	7.93^abc^ ± 1.32	9.88^ab^ ± 1.44
Energy (2600)–protein (24)		1.12^a^ ± 0.13	1.53^b^ ± 0.10	2.40^c^ ± 0.19	3.44^b^ ± 0.26	4.17^b^ ± 0.27	5.69^c^ ± 0.84	6.80^c^ ± 0.64	8.60^b^ ± 0.60

Means (± standard error) within each column of dietary treatments with no common superscript differ significantly at *P* < 0.05

**Table 3:** Mean body size (±SE) of ostrich chicks in the 2nd and 9th week of age fed with diets containing the different levels of energy and protein (cm)

Time (weeks)	Dietary treatment		Body characteristics											
			Beak circumference	Head circumference	Neck height	Breast circumference	Abdomen circumference	Thigh circumference	Thigh length	Leg length	Arm circumference	Arm length	Body length	Body height
2	Energy (kcal/kg DM)	2400	3.42^a^ ± 0.22	14.38^a^ ± 0.17	15.64^a^ ± 1.31	23.30^a^ ± 2.84	28.08^a^ ± 1.15	20.22^a^ ± 4.23	7.95^a^ ± 0.47	8.58^a^ ± 0.31	4.73^a^ ± 0.57	4.84^a^ ± 0.88	56.63^a^ ± 2.25	25.74^a^ ± 1.61
		2600	3.49^a^ ± 0.30	14.16^a^ ± 0.27	15.61^a^ ± 0.99	20.33^a^ ± 1.74	26.58^b^ ± 1.21	21.27^a^ ± 2.77	7.30^b^ ± 0.32	7.91^b^ ± 0.83	4.41^a^ ± 0.51	5.45^a^ ± 0.61	55.53^a^ ± 1.86	25.30^a^ ± 1.22
	Protein (% in diet)	20	3.53^a^ ± 0.32	14.33^a^ ± 0.26	14.90^a^ ± 0.69	21.30^a^ ± 1.82	27.31^a^ ± 1.05	19.32^a^ ± 3.27	7.24^b^ ± 0.28	4.48^a^ ± 0.48	4.37^a^ ± 0.46	4.94^a^ ± 0.94	55.84^a^ ± 2.21	25.23^a^ ± 1.26
		22	3.37^a^ ± 0.21	14.18^a^ ± 0.31	16.01^a^ ± 1.53	21.53^a^ ± 4.09	27.77^a^ ± 1.57	21.75^a^ ± 3.56	7.62^ab^ ± 0.41	7.68^b^ ± 0.99	4.42^a^ ± 0.74	5.39^a^ ± 0.70	55.81^a^ ± 2.35	25.32^a^ ± 1.74
		24	3.49^a^ ± 0.29	14.23^a^ ± 0.23	15.94^a^ ± 0.57	21.72^a^ ± 1.97	26.46^a^ ± 1.40	21.49^a^ ± 3.31	7.83^a^ ± 0.63	8.38^a^ ± 0.47	4.84^a^ ± 0.32	5.30^a^ ± 0.73	56.27^a^ ± 1.94	25.88^a^ ± 1.20
	Energy (2400)–protein (20)		3.30^a^ ± 0.28	14.46^a^ ± 0.30	14.89^a^ ± 1.05	22.50^a^ ± 2.82	28.28^a^ ± 1.06	18.80^a^ ± 2.40	7.47^bc^ ± 0.31	8.20^a^ ± 0.02	4.55^a^ ± 0.78	4.48^a^ ± 0.67	54.72^a^ ± 1.06	24.58^a^ ± 1.68
	Energy (2400)–protein (22)		3.56^a^ ± 0.16	14.34^a^ ± 0.12	16.54^a^ ± 2.06	24.46^a^ ± 4.54	28.80^a^ ± 0.28	21.89^a^ ± 6.20	7.93^ab^ ± 0.26	8.70^a^ ± 0.06	4.55^a^ ± 0.78	5.22^a^ ± 1.23	57.61^a^ ± 1.96	26.19^a^ ± 1.14
	Energy (2400)–protein (24)		3.40^a^ ± 0.28	14.34^a^ ± 0.12	15.49^a^ ± 0.71	22.93^a^ ± 2.73	27.16^a^ ± 1.64	19.98^a^ ± 5.98	8.45^a^ ± 0.06	8.83^a^ ± 0.24	5.10^a^ ± 0.14	4.84^a^ ± 1.18	57.57^a^ ± 3.08	26.47^a^ ± 2.16
	Energy (2600)–protein (20)		3.68^a^ ± 0.29	14.25^a^ ± 0.25	14.91^a^ ± 0.62	20.50^a^ ± 0.50	26.66^a^ ± 0.28	19.66^a^ ± 4.25	7.08^c^ ± 0.14	8.66^a^ ± 0.57	4.25^a^ ± 0.25	5.25^a^ ± 1.08	56.58^a^ ± 2.67	25.66^a^ ± 1.04
	Energy (2600)–protein (22)		3.25^a^ ± 0.13	14.08^a^ ± 0.38	15.66^a^ ± 1.46	19.58^a^ ± 2.98	27.08^a^ ± 1.77	21.66^a^ ± 2.46	7.41^bc^ ± 0.38	7.00^b^ ± 0.50	4.33^a^ ± 0.87	5.50^a^ ± 0.43	54.61^a^ ± 1.95	24.75^a^ ± 2.04
	Energy (2600)–protein (24)		3.55^a^ ± 0.35	14.16^a^ ± 0.28	16.25^a^ ± 0.25	20.91^a^ ± 1.28	26.00^a^ ± 1.32	22.50^a^ ± 0.50	7.41^bc^ ± 0.38	8.08^a^ ± 0.28	4.66^a^ ± 0.28	5.61^a^ ± 0.12	55.41^a^ ± 0.14	25.50^a^ ± 0.00
9	Energy (kcal/kg DM)	2400	6.34^a^ ± 0.34	21.16^a^ ± 0.39	48.27^a^ ± 2.67	50.47^a^ ± 4.54	60.72^a^ ± 2.46	53.44^a^ ± 2.09	16.63^a^ ± 0.77	24.13^a^ ± 1.13	13.97^a^ ± 0.98	17.36^a^ ± 1.17	134.13^a^ ± 3.49	66.50^a^ ± 2.88
		2600	6.03^b^ ± 0.18	20.78^a^ ± 0.61	45.61^a^ ± 2.70	46.43^b^ ± 1.84	56.11^b^ ± 2.42	49.76^b^ ± 1.71	15.43^b^ ± 0.82	23.65^a^ ± 1.18	13.61^a^ ± 0.65	17.38^a^ ± 0.61	132.53^a^ ± 4.64	65.12^a^ ± 2.8
	Protein (% in diet)	20	6.13^a^ ± 0.26	20.95^ab^ ± 0.64	46.00^a^ ± 3.45	50.04^a^ ± 4.34	57.91^a^ ± 3.70	52.41^a^ ± 2.74	16.04^a^ ± 1.12	23.41^a^ ± 0.60	13.41^a^ ± 0.58	17.04^a^ ± 0.62	134.30^a^ ± 4.19	64.75^a^ ± 2.01
		22	6.10^a^ ± 0.22	21.35^a^ ± 0.48	47.80^a^ ± 2.25	47.20^a^ ± 2.97	60.30^a^ ± 3.09	51.40^a^ ± 1.81	16.35^a^ ± 0.78	24.15^a^ ± 1.38	14.00^a^ ± 0.84	17.25^a^ ± 0.58	131.90^a^ ± 4.10	65.40^a^ ± 3.66
		24	6.35^a^ ± 0.40	20.70^b^ ± 0.29	47.39^a^ ± 3.12	48.25^a^ ± 4.59	57.73^a^ ± 3.19	51.26^a^ ± 3.39	15.87^a^ ± 1.11	24.20^a^ ± 1.36	14.03^a^ ± 1.03	17.80^a^ ± 1.30	133.18^a^ ± 4.66	67.33^a^ ± 2.63
	Energy (2400)–protein (20)		6.16^b^ ± 0.38	21.41^ab^ ± 0.38	47.58^a^ ± 3.00	53.08^a^ ± 3.84	60.66^ab^ ± 1.89	54.83^a^ ± 1.04	16.91^a^ ± 0.52	23.58^a^ ± 0.80	13.33^a^ ± 0.76	16.75^a^ ± 0.25	134.01^a^ ± 4.96	65.50^a^ ± 1.80
	Energy (2400)–protein (22)		6.16^b^ ± 0.16	21.25^abc^ ± 0.43	48.33^a^ ± 2.25	47.50^ab^ ± 3.77	61.83^a^ ± 2.02	51.66^abc^ ± 0.28	16.25^abc^ ± 1.08	24.08^a^ ± 0.87	14.41^a^ ± 0.14	17.08^a^ ± 0.72	131.55^a^ ± 2.19	64.83^a^ ± 2.75
	Energy (2400)–protein (24)		6.70^a^ ± 0.15	20.83^abc^ ± 0.14	48.91^a^ ± 3.64	50.83^ab^ ± 5.48	59.66^abc^ ± 3.61	53.83^ab^ ± 2.92	16.75^a^ ± 0.75	24.75^a^ ± 1.63	14.16^a^ ± 1.52	18.25^a^ ± 1.75	136.84^a^ ± 1.89	69.16^a^ ± 2.46
	Energy (2600)–protein (20)		6.10^b^ ± 0.18	20.50^c^ ± 0.50	44.41^a^ ± 3.64	47.00^ab^ ± 2.17	55.16^c^ ± 2.84	50.00^c^ ± 0.50	15.16^bc^ ± 0.76	23.25^a^ ± 0.43	13.50^a^ ± 0.50	17.33^a^ ± 0.80	134.50^a^ ± 4.76	64.00^a^ ± 2.29
	Energy (2600)–protein (22)		6.00^b^ ± 0.35	21.50^a^ ± 0.70	47.00^a^ ± 2.82	46.75^ab^ ± 2.47	58.00^abc^ ± 3.53	51.00^bc^ ± 3.53	16.50^ab^ ± 0.00	24.25^a^ ± 2.47	13.37^a^ ± 1.23	17.50^a^ ± 0.35	132.25^a^ ± 6.71	66.25^a^ ± 6.01
	Energy (2600)–protein (24)		6.00^b^ ± 0.10	20.58^bc^ ± 0.38	45.88^a^ ± 2.06	45.66^b^ ± 1.60	55.79^bc^ ± 1.12	48.70^c^ ± 0.71	15.00^c^ ± 0.50	23.66^a^ ± 1.04	13.89^a^ ± 0.53	17.36^a^ ± 0.77	130.75^a^ ± 4.42	65.50^a^ ± 1.09

Means (± standard error) within each column of dietary treatments with no common superscript differ significantly at *P* < 0.05

**Table 4:** Excretion microbial population mean (±SE) of ostrich chicks at 9th week of age fed with diets containing the different levels of energy and protein from the 2nd–9th weeks of age (CFU/g)

		Trait			
Treatment		*Escherichia coli*	Coliforms bacteria	Lactobacillus bacteria	Aerobic bacteria total
Energy (kcal/kg DM)	2400	8.66^a^ ± 0.74	9.07^a^ ± 0.70	8.54^a^ ± 0.76	10.30^a^ ± 0.85
	2600	8.37^a^ ± 0.72	8.93^a^ ± 1.16	6.87^b^ ± 1.37	9.53^a^ ± 1.05
Protein (% in diet)	20	8.24^a^ ± 0.29	8.66^a^ ± 0.50	7.84^a^ ± 1.50	10.21^a^ ± 1.01
	22	8.63^a^ ± 1.04	9.20^a^ ± 1.43	7.22^a^ ± 1.66	10.07^a^ ± 1.24
	24	8.68^a^ ± 0.70	9.14^a^ ± 0.70	8.06^a^ ± 0.98	9.47^a^ ± 0.72
Energy (2400)–protein (20)		8.28^a^ ± 0.13	8.95^a^ ± 0.47	8.75^a^ ± 0.56	10.45^ab^ ± 0.75
Energy (2400)–protein (22)		8.81^a^ ± 1.15	8.99^a^ ± 1.22	8.19^ab^ ± 1.30	11.04^a^ ± 0.19
Energy (2400)–protein (24)		8.89^a^ ± 0.73	9.28^a^ ± 0.44	8.69^a^ ± 0.16	9.42^ab^ ± 0.53
Energy (2600)–protein (20)		8.20^a^ ± 0.43	8.36^a^ ± 0.38	6.93^ab^ ± 1.69	9.98^ab^ ± 1.34
Energy (2600)–protein (22)		8.44^a^ ± 1.12	9.41^a^ ± 1.86	6.25^b^ ± 1.55	9.10^b^ ± 1.00
Energy (2600)–protein (24)		8.46^a^ ± 0.74	9.00^a^ ± 0.98	7.43^ab^ ± 1.10	9.51^ab^ ± 1.01

Means (± standard error) within each column of dietary treatments with no common superscript differ significantly at *P* < 0.05

## Discussion

In this study, we have defined how different levels of energy and protein affect body weight, development of particular body parts, and microflora in young ostriches. The obtained results indicated that increasing the energy and protein level decreased body weight gain. Similarly, Mahrose et al. (2015) showed that both initial and final live weight and body weight gain of ostrich chickens during 2–9 weeks of age were not significantly affected by dietary protein level. In these studies, the highest body weight was observed in the case of the lowest level of protein. In turn, Tasirnafas et al. (2015) revealed that higher body weight was achieved by feeding lower dietary energy levels.

The present study monitored the dynamics of body parameters from the 2nd to 9th weeks of age. Young, growing ostriches should be healthy and in good condition. In the early months of life, it is important to develop key statural parameters. The most important parameters including the breast, abdomen, and thigh circumference and also body length were higher in birds offered the lower energy level (2400 kcal/kg). However, greater tail length and shank circumference were observed in birds fed with the higher energy level (2600 kcal/kg). Results of the present study indicated that ostrich chickens during 2–9 weeks of age could grow efficiently on diets that contain lower energy levels. The effect of dietary protein levels on body height, tibiotarsus length, and tibiotarsus girth has also been reported by Mahrose et al. (2015). In their study, only tibiotarsus girth was decreased with increasing dietary protein, while body height and tibiotarsus length were not significantly affected. In our case, the dietary protein level did not significantly affect the studied body characteristics. Generally, the values of most traits were a little higher in birds fed with the highest protein level (24%). In a previous study, Carstens et al. (2014) evaluated the growth response of ostrich chicks to diets containing different concentrations of protein. These authors reported no significant differences in weight gain from 1 to 49 days of age between birds fed with high and low protein diets, and no significant differences in weight gain between birds fed with the high and medium protein levels from 1 to 77 days of age. Similarly, no significance in weight gain was observed in birds fed with the medium and low protein level from 1 to 98 days of age. We also measured the mean excretion of enteric microbial populations in ostrich chickens. Little has been reported on the nature of enteric ostrich microflora. Young birds can be affected by *Escherichia coli*, *Campylobacter*, and *Salmonella* bacteria. *Escherichia coli* occurs naturally in the ostrich’s intestine, but some of its strains may be pathogenic. *Campylobacter* and *Salmonella* are pathogenic and occur in ostriches and become a potential risk to humans. Every breeder aims to keep healthy birds, and therefore the assessment of microflora in the gastrointestinal tract is very important. We determined the occurrence of *Escherichia coli*, *Coliforms*, *Lactobacillus*, and total *Aerobic* bacteria in ostriches. Our results showed that total *Aerobic* bacteria were generally lower in birds fed with the diet containing higher levels of energy (2600 kcal/kg).

## Conclusion

Our results provide quantitative information on the impact of dietary energy and protein on body weight, body size and excretion microbial population. The increase the level of energy showed depressive effect on the body weight and body size. However, further studies are needed to confirm these results so that the growth performance of ostrich can be enhanced at minimal cost.
